# An Evidence-Based Retrospective Study for the Management of Acid Peptic Disease With Omeprazole, a Proton Pump Inhibitor, in Indian Patients With Type 2 Diabetes Mellitus (PRIDE-1)

**DOI:** 10.7759/cureus.32332

**Published:** 2022-12-08

**Authors:** Bharat Saboo, Nimmi Mulwani, Anup U Petare, Krishna C Veligandla, Colette S Pinto, Amey Mane, Rahul Rathod, Bhavesh Kotak

**Affiliations:** 1 Department of Diabetology, Prayas Diabetes Centre, Indore, IND; 2 Department of Diabetology, Dr. Nimmi’s Diabetes Care, Ahmedabad, IND; 3 Medical Affairs, Dr. Reddy’s Laboratories Ltd., Hyderabad, IND

**Keywords:** type 2 diabetes mellitus, symptomatic relief, proton pump inhibitor, electronic medical record, acid peptic disease

## Abstract

Background

In this study, we aimed to assess the effectiveness of omeprazole therapy in the management of acid peptic disease (APD) among type 2 diabetes mellitus (T2DM) patients.

Methodology

In this multicenter retrospective study, electronic medical records (EMRs) of T2DM patients with APD who were prescribed omeprazole between March 2018 and April 2021 at multiple Indian healthcare settings were reviewed. The resolution of APD symptoms was assessed at visit five (120 days after the index visit) and compared to visit one (index visit). Safety was established in terms of reported adverse events during the study period.

Results

Overall, 174 patients were included. The majority of patients (63.8%) were males with a mean age of 48.6 ± 11.03 years. After receiving omeprazole therapy, a significant number of patients reported improvement in symptoms such as abdominal pain (98.2%), epigastric burning (74.2%), altered bowel movements (62.1%), and nausea (80.5%) (p < 0.001 for each). Complete resolution was observed in all patients who complained about flatulence (100.0%) and loss of appetite (100.0%) (p < 0.001 for each). The drug was found to be well tolerated.

Conclusions

Omeprazole therapy was well tolerated and highly effective in resolving APD symptoms among T2DM patients receiving fixed oral hypoglycemic agents.

## Introduction

Diabetes mellitus (DM) is a heterogeneous metabolic disorder characterized by hyperglycemia, depletion of antioxidants, and lipid metabolism changes [1]. The resultant augmentation of reactive oxygen species (ROS), motor dysfunction, and enteric neuronal loss lead to motility disturbances due to irreversible autonomic neuropathy; hence, an increase in the incidence of gastrointestinal tract (GIT) symptoms in DM patients [2]. Recent clinical studies have reported the prevalence of esophageal dysmotility and gastroesophageal reflux (GERD) symptoms in DM patients to be as high as 63% and 41%, respectively [3,4]. All of these factors contribute to the development of acid peptic diseases (APD) in DM patients. APD is a collective term that includes gastrointestinal conditions such as GERD, peptic ulcer disease, gastritis, Zollinger-Ellison syndrome, and Meckel’s diverticulum ulcer [5]. It is a consequence of distinctive yet imbricated pathogenic mechanisms leading to either enhanced acid secretion or weakened mucosal defense [6]. These acid-related diseases remarkably impact patients’ quality of life. Patients usually complain about acid reflux, regurgitation, chest pain, cough, and dysphagia [7]. Other frequent symptoms include headaches, dizziness, diarrhea, abdominal pain, constipation, nausea, vomiting, and flatulence [5,8].

Proton pump inhibitors (PPIs) are the cornerstone of APD treatment and are easily available on prescription as well as over the counter [9]. They are believed to act by inhibiting the hydrogen-potassium ATPase (H+/K+-ATPase) pump (located in the gastric parietal cells) which is responsible for hydrochloric acid (HCl) secretion into the gastric lumen. PPIs bind covalently with the H+/K+-ATPase pump, ensuing an irreversible inhibition of HCl secretion [10]. Among all PPIs, omeprazole was first marketed, and it has been observed to be a very potent inhibitor of gastric acid secretion, with a long-lasting duration of action [11]. The effectiveness of omeprazole in providing symptomatic relief to APD patients is also well-recognized [12]. Apart from managing gastric complications associated with diabetes, PPIs have also demonstrated their role in improving glycemic control, possibly by augmenting both serum levels of gastrin and β cell mass [13].

Despite the common usage of PPIs among APD patients, there are limited studies evaluating the effectiveness and safety of these agents among DM patients in Indian settings. Therefore, the present study (PRIDE-1) intends to provide real-world, data-based insights into the effectiveness and safety of omeprazole therapy in managing APD among type 2 diabetes mellitus (T2DM) patients receiving fixed antidiabetic therapy.

## Materials and methods

This was a retrospective, observational study conducted by collecting data from electronic medical records (EMRs) of T2DM patients with APD visiting outpatient settings of Indian tertiary healthcare centers between March 2018 and April 2021.

Study population

Patients aged ≥18 years with T2DM who were newly diagnosed with APD (diabetic patients who were diagnosed with APD for the first time at the index visit) and receiving fixed oral hypoglycemic agents (OHAs) for a minimum of three months before visit 1 (index visit) were included in the study. Patients with the same fixed antidiabetic medications and receiving omeprazole (20 mg) for APD for a minimum of four weeks after the index visit were included in the study. However, patients with type 1 DM or gestational DM, those on insulin or other injectables, and T2DM patients treated with H2-receptor antagonists or other PPIs for APD treatment were excluded from the study.

Study outcomes

The study outcomes were evaluated at visit 0 (90 days before visit 1), visit 1 (index visit), and visits 2, 3, 4, and 5 (i.e., 30, 60, 90, and 120 days after the index visit). The primary outcome of the study was to determine the clinical improvement, defined as symptomatic relief or resolution of symptoms, i.e., pain in the abdomen, epigastric burning, nausea, flatulence, loss of appetite, and altered bowel movements, etc., at visit 5 compared to visit 1 in APD patients with T2DM receiving omeprazole. Further, safety was assessed in terms of adverse events (AEs) reported during the study period. The secondary outcome of the study was to assess the effect of omeprazole on glycemic control, which was determined as the mean percentage decrease in glycemic parameters (e.g., HbA1c, fasting blood sugar (FBS), post-prandial blood sugar (PPBS), and random blood sugar (RBS)) at visit 1 compared to visit 0 and at visit 5 compared to visit 1.

Statistical analysis

Data were analyzed using R studio 1.2.1335. Continuous variables such as age and duration were presented as means ± standard deviation (SD) and compared using the t-test/Mann-Whitney U test. Categorical variables such as gender and city/state were presented as percentages/proportions and compared using the chi-square test/Fisher’s exact test. Statistical significance was considered at p-values <0.05.

## Results

Baseline characteristics

A total of 174 patients with T2DM and APD were included in the study. Overall, the majority of patients were males, i.e., 111 (63.8%) with a mean age of 48.6 ± 11.0 years. Other parameters such as weight, pulse, body temperature, and systolic and diastolic blood pressures are tabulated in Table 1. Several patients were found to have multiple comorbidities, with cardiovascular comorbidity (28.7%) being the most frequently reported. Hypertension was the most reported condition (94.0%) among 50 patients with cardiovascular comorbidities. Further, cardiovascular drugs (78.0%) were the most commonly used concomitant drugs, followed by drugs for endocrine disorders (19.3%). Few patients were also found to receive diuretics (0.9%), drugs for respiratory disorders (0.9%), and anti-inflammatory drugs (0.9%). Among OHAs, the majority of patients (52.3%) were found to receive the combination of metformin and glimepiride, followed by metformin and vildagliptin/sitagliptin (28.2%), and metformin monotherapy (19.5%) (Table 1).

**Table 1:** Baseline characteristics of patients included in the study.

Parameter	Variable (N = 174)
Age (years) (mean ± SD)	48.64 ± 11.03
Height (cm) (mean ± SD)	166.07 ± 9.73
Weight (kg) (mean ± SD)	74.51 ± 13.47
Pulse (beats per minute) (mean ± SD)	77.94 ± 6.66
Body temperature (°F) (mean ± SD)	97.96 ± 0.46
Systolic blood pressure (mmHg) (mean ± SD)	126.54 ± 10.45
Diastolic blood pressure (mmHg) (mean ± SD)	77.81 ± 7.55
Gender	
Female	63 (36.2%)
Male	111 (63.8%)
State	
Gujarat	111 (63.8%)
Maharashtra	27 (15.5%)
Madhya Pradesh	36 (20.7%)
Oral hypoglycemic agents	
Metformin + glimepiride	91 (52.3%)
Metformin + vildagliptin/sitagliptin	49 (28.2%)
Metformin	34 (19.5%)
Comorbidities	
Cardiovascular	50 (28.7%)
Neurological	1 (0.6%)
Renal	2 (1.1%)
Respiratory	2 (1.1%)
Orthopedic	1 (0.6%)
Others	39 (22.4%)
Concomitant medications	
Cardiovascular	85 (78.0%)
Endocrine	21 (19.3%)
Respiratory	1 (0.9%)
Anti-inflammatory	1 (0.9%)
Diuretic	1 (0.9%)

Effectiveness

Symptomatic Relief

The effectiveness of omeprazole was assessed in terms of the number of patients who reported relief at visit 5 compared to visit 1. At visit 1, abdominal pain (64.9%) and epigastric burning (55.7%) were the chief complaints, followed by altered bowel movements (38.0%), nausea (23.6%), loss of appetite (21.3%), and flatulence (16.6%).

**Figure 1:**
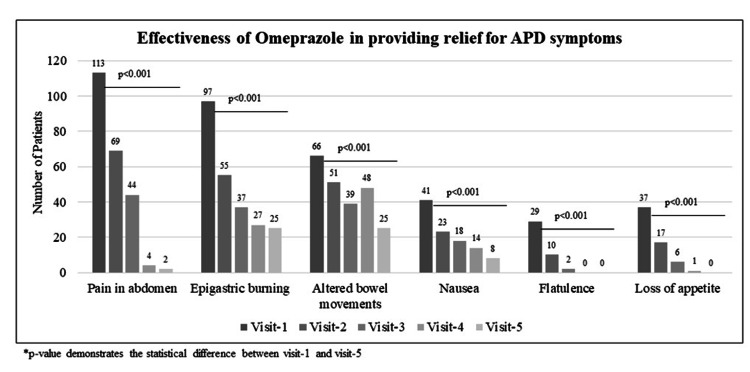
Effectiveness of omeprazole in providing symptomatic relief to acid peptic disease (APD) patients.

The number of patients with abdominal pain at visit 1 (n = 113) was significantly reduced at visit 5 (n = 2), implying that 98.2% of patients had symptomatic relief (p < 0.001). Further, epigastric burning was reported by 97 patients at visit 1, and the number gradually reduced to 25 at visit 5, wherein 74.2% of patients had symptomatic relief (p < 0.001). Improvement in altered bowel movements was reported by 62.1% of patients (the number of patients complaining about the symptom decreased from 66 to 25) (p < 0.001). The patients who reported nausea declined from 41 (at visit 1) to eight (at visit 5), demonstrating that 80.5% of patients got relief from nausea (p < 0.001). Complete resolution was observed in all (100%) patients who complained about flatulence (p < 0.001) as well as loss of appetite (p < 0.001) at visit 5 (Figure 1).

Glycemic control

**Figure 2:**
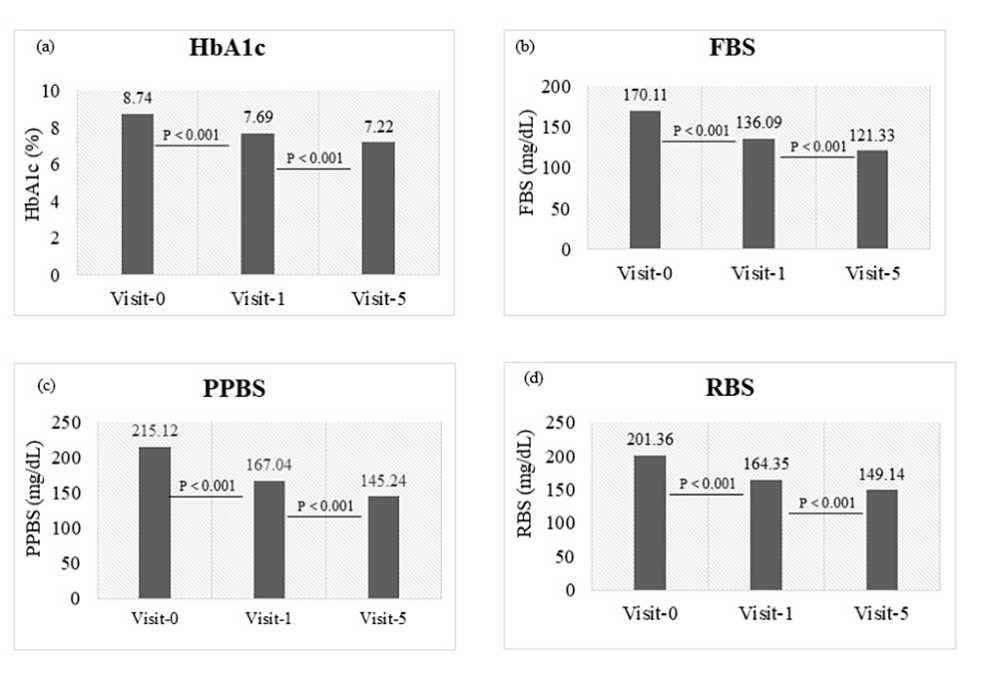
Effectiveness of omeprazole in mean reduction in (a) glycated hemoglobin (HbA1c), (b) fasting blood sugar (FBS), (c) post-prandial blood sugar (PPBS), and (d) random blood sugar (RBS).

The percentage reduction in mean HbA1c value was observed to be 12.0% (from visit 0 to 1) while the patients were taking only OHAs (p < 0.001). A further significant reduction of 6.1% was observed while patients were taking OHAs along with omeprazole (i.e., from visit 1 to 5; p < 0.001). Similarly, the percentage reduction in the mean FBS level was found to be 19.9% (p < 0.001) from visit 0 to 1, with a reduction of 10.8% from visit 1 to 5 (p < 0.001). In the case of mean PPBS, a marked reduction of 22.4% was observed from visit 0 to 1 (p < 0.001), with a subsequent reduction of 13.1% from visit 1 to 5 (p < 0.001). The decrease in mean RBS levels was noted to be 18.3% (from visit 0 to 1; p < 0.001), followed by a reduction of 9.2% (from visit 1 to 5; p < 0.001) (Figure 2).

Tolerability

Omeprazole was found to be well tolerated in T2DM patients with APD as none of the patients complained of AEs during the study period.

## Discussion

T2DM is associated with an escalated prevalence of upper and lower gastrointestinal complications. PPIs have emerged as the preferred therapy for various acid peptic disorders, and, thus, the present study evaluated the real-world evidence of omeprazole in the management of APD among Indian patients with T2DM.

The effectiveness analysis of the PRIDE-1 study revealed that omeprazole therapy effectively provides symptomatic relief to T2DM patients with APD. Literature has also evidenced that omeprazole is well-tolerated and effective in acid-related diseases. In a randomized, double-blinded, placebo-controlled trial, omeprazole was observed to provide significant relief from heartburn in GERD patients (n = 359) compared to placebo (62% vs. 14%; p ≤ 0.002) after seven days of treatment. Moreover, omeprazole was found to be significantly (p ≤ 0.003) more effective than placebo for the management of acid regurgitation, dysphagia, epigastric pain, and nausea [14]. Similarly, in another study, after a treatment period of four weeks, a significantly greater number of patients (n = 209) receiving omeprazole compared to placebo reported relief from reflux symptoms such as heartburn (57% vs. 19%; p < 0.0001) and regurgitation (75% vs. 47%; p < 0.0001). Further, a higher number of patients in the omeprazole group were completely asymptomatic (43%) compared to the placebo group (p < 0.0001) [15]. A review analyzing the results of large, randomized, double-blinded clinical trials (BOND, OPERA, PILOT, and ENCORE) also summarized that complete symptom relief was achieved in a higher proportion of dyspepsia patients treated with omeprazole (38.2% with 20 mg; 36.0% with 10 mg) (p < 0.05) compared to placebo (28.2%) [16].

In the PRIDE-1 study, omeprazole, when administered along with OHAs, was found to be relatively safe as no major AE (requiring specific medical intervention) was observed. This is consistent with the existing literature that states PPIs are relatively safe medications [17]. The risk of minor AEs (requiring no specific medical intervention) from PPIs is reported to be relatively low (1-3%), and the incidence of serious AEs is stated to be rare. In a meta-analysis of published trials involving 2,812 patients, omeprazole was reported to cause headache (2.4%), diarrhea (1.9%), nausea (0.9%), and rash (1.1%) and was reported to be a relatively safe drug [17].

Further, in this study, a significant improvement in glycemic parameters was observed in patients after taking OHAs (from visit 0 to visit 1). A subsequent marked reduction in glycemic parameters observed from visit 1 to 5 might be attributed to the addition of omeprazole to the existing combination of OHAs. The role of PPI in glycemic control in T2DM patients has been elucidated by their action of lowering gastric acid, subsequently increasing gastrin levels that stimulate beta-cell proliferation and function [18]. Chandra et al. (2018) observed a marked reduction in HbA1c, FBS, and PPBS levels in T2DM patients after receiving PPI for 24 weeks [19]. Another study reported significant improvement in glycemic parameters (FBS: 108 ± 2.37 vs. 126 ± 2.9; HbA1c: 7.29 ± 0.07 vs. 7.47 ± 0.04) with omeprazole for 12 weeks along with antidiabetic medication compared to patients taking antidiabetic medications alone [20]. Similarly, a retrospective survey of a clinical database of DM patients revealed that patients taking concurrent PPIs (n = 282) had significantly lower values of average HbA1c (7.0% ) compared to patients (n = 65) who had not received PPIs (7.6%) [21]. Although some studies have reported an improvement in glycemic parameters with the use of PPIs, their clinical effect on glycemic control is not yet fully established. Therefore, randomized controlled studies of PPIs with a more significant number of T2DM patients are warranted to corroborate the effect of PPIs on glycemic control.

Limitations

The study was retrospective in nature, due to which the analysis was limited to the information available in existing database records. Further, due to the retrospective design of the study, it lacks the presence of a control group which would have facilitated precise differentiation between the impact of OHAs and omeprazole on APD patients. Additionally, certain symptoms induced by concomitant medications cannot be differentiated from the presenting symptoms of APD.

## Conclusions

Omeprazole has been widely used to prevent and manage several acid-related disorders. The PRIDE-1 study demonstrated its significant effectiveness in providing symptomatic relief in T2DM patients with APD. Further, omeprazole was found to be well-tolerated among DM patients taking fixed OHAs. It may also potentiate glycemic control in these patients, which needs to be further validated via well-controlled randomized studies.
